# Diagnosis and surgical management of septic peritonitis in small animals: A review

**DOI:** 10.1111/vsu.70024

**Published:** 2025-10-18

**Authors:** Bonnie G. Campbell, Shana K. O'Marra

**Affiliations:** ^1^ Department of Veterinary Clinical Sciences, College of Veterinary Medicine Washington State University Pullman Washington USA

## Abstract

**Background:**

Septic peritonitis (SP) is a complex disease with significant morbidity and mortality. Diagnosis can be challenging, and lack of a timely diagnosis may impact survival.

**Aim:**

The aim of this review was to synthesize current knowledge on the diagnosis and surgical management of SP in dogs and cats.

**Conclusions:**

Recent studies suggest refinements of previously reported clinicopathologic criteria improve diagnostic and prognostic accuracy. When evaluating blood‐effusion glucose differences, clinicians should be aware of the confounding effects of point of care glucometers. Cytology specimens should be prepared immediately to avoid sample degradation. Advances in imaging have improved the relevance and diagnostic value of ultrasound and computed tomography. Surgical source control that targets the underlying cause is crucial. Accurate assessment of tissue viability may be aided by technologies under development. Anastomotic dehiscence may be reduced by using stapling devices and reinforcement with endogenous or exogenous materials. Evidence for the effectiveness of postoperative drainage via open abdomen ± vacuum‐assisted closure, or closed suction drains, is unclear. Heterogeneity of patient severity and incomplete data limit direct comparisons between studies and highlight opportunities for future investigation.

**Implications:**

Developments in diagnostic and surgical techniques may improve prognostication and outcomes in septic patients. There are many opportunities for further research into diagnosis and surgical treatment of patients with septic peritonitis.

## 1 INTRODUCTION

Septic peritonitis (SP) is a life‐threatening condition caused by microbial contamination of the peritoneal cavity, most commonly secondary to gastrointestinal perforation. In studies that extended at least into the last 15 years, survival rates for dogs and cats with SP of any cause ranged from 36.4% to 100%.^1^, ^2^, ^3^, ^4^, ^5^, ^6^, ^7^, ^8^, ^9^, ^10^, ^11^, ^12^, ^13^, ^14^, ^15^, ^16^, ^17^, ^18^, ^19^, ^20^, ^21^, ^22^, ^23^, ^24^, ^25^, ^26^ The gastrointestinal tract (GIT) was the source in >75% of cases, with survival rates of 36.4%–88.5%.^1^, ^2^, ^3^, ^4^, ^12^, ^16^, ^17^, ^18^, ^19^, ^21^, ^24^, ^25^, ^26^, ^27^ Survival rates for dogs and cats developing recurrent SP were 0%–43.9%.^12^, ^15^, ^18^, ^26^, ^27^, ^28^, ^29^, ^30^, ^31^ Sepsis definitions in humans have evolved to better identify patients that benefit from early intervention, with similar efforts underway in veterinary medicine.^32^, ^33^ Improved diagnostic methods may allow earlier diagnosis, targeted therapy, and better prognostication, and advances in surgical techniques may reduce complications. The aim of this review was to report recent advances in identification and surgical management of SP in small animals and highlight research opportunities. Perioperative management of the SP patient is reviewed in a companion paper.

## 2 DIAGNOSTIC INVESTIGATION

Clinical signs in dogs include lethargy, anorexia, vomiting, diarrhea, dehydration, fever, abdominal pain, abdominal effusion, and shock. Cats often show subtler signs, delaying diagnosis; temperature and heart rate can be increased or decreased,^6^, ^34^, ^35^ and abdominal pain may be absent.^34^ Early achievement of hemodynamic goals and timely antimicrobial therapy predict outcome in human septic patients,^36^ and should be initiated during diagnostic testing.

### 2.1 Bloodwork

Cageside tests like packed cell volume, total solids, glucose, lactate and venous blood gas aid in determining initial stabilization steps.

Leukocytosis, leukopenia, degenerate left shift (DLS) and toxic neutrophils are common in SP; however, their prognostic value has been inconsistent for dogs and cats. A 2013 study found that SP dogs with DLS were twice as likely to die as those without.^37^ A subsequent study found no association between DLS and mortality in dogs with sepsis, including a subgroup of 11 SP dogs, although non‐survivors had lower neutrophil counts.^38^ Toxic change (but not total WBC, bands, lymphocyte or neutrophil counts) was associated with increased mortality in dogs undergoing large bowel surgery.^39^ Other studies in dogs and cats with SP found no difference in WBC,^4^, ^34^ neutrophils,^40^ or band neutrophils between survivors and non‐survivors,^40^ or between those that did or did not develop acute kidney injury (AKI).^41^ Similarly, neutrophil to lymphocyte ratio did not differentiate SP dogs from those with non‐septic inflammation, or predict survival.^22^

Platelet counts may be increased or decreased but have not been associated with mortality in multiple studies of SP dogs.^41^, ^42^, ^43^, ^44^, ^45^ Elevated mean platelet volume was associated with mortality in a pilot study of platelet indices of SP dogs.^41^

A biochemistry panel provides important diagnostic and prognostic information that impacts treatment. Electrolyte derangements and hypoalbuminemia may inform fluid therapy choices, and pre‐existing or acquired hepatic and renal dysfunction impact drug metabolism and clearance. Relevant biochemical variables are described in Table 1.

**TABLE 1:** Common serum biochemistry abnormalities in patients with SP.

Abnormality	Mechanisms	Prognostic value	Comments
↓Glucose	↓Intake ↓Hepatic function ↑Utilization	Not associated with mortality in dogs or cats^5^, ^6^, ^34^, ^42^, ^46^, ^47^ Increased mortality in dogs undergoing large intestinal surgery^39^	
↑Glucose (cats)	Multifactorial,^5^ may involve: Stress hyperglycemia ↓Glycogen synthesis ↓Insulin ↓Insulin: glucagon	Increased mortality in cats^5^	Not specifically reported in dogs
↑Lactate	Tissue hypoperfusion Mitochondrial dysfunction	Preoperative ↑lactate associated with mortality in dogs^3^, ^4^ Failure to normalize lactate postoperatively strongly associated with mortality in dogs^3^	
↓Albumin	Vascular, peritoneal leak Hepatic dysfunction Acute phase shift in protein production ↓Nutrition	*Preoperative*: Risk of dehiscence after gastrointestinal surgery: Increased (dogs, cats)^15^, ^48^, ^49^, ^50^ No association (dogs, cats)^4^, ^26^, ^28^, ^29^, ^39^, ^51^, ^52^, ^53^, ^54^, ^55^, ^56^, ^57^ Risk of mortality or recurrence of SP: Increased (dogs)^4^, ^15^, ^24^, ^42^ No association (dogs, cats)^5^, ^6^, ^25^, ^40^, ^52^, ^58^, ^59^, ^60^ *Postoperative*: Severe hypoalbuminemia increases mortality risk in dogs^4^, ^61^	Functions^62^, ^63^: ↑Oncotic pressure Buffer Carrier of hormones, drugs, toxins, ions Antioxidant Indirect role in wound healing Vasodilation ↓Platelet aggregability
↑Liver values	Impaired perfusion Sepsis‐associated cholestasis Cellular injury^64^, ^65^	↑Alkaline transferase ↑Mortality (dogs, cats)^34^, ^66^ No association (dogs)^4^ ↑Alkaline phosphatase ↑Mortality (dogs)^4^ No association (dogs, cats)^34^, ^66^ ↑Gamma‐glutamyl transferase ↑Mortality (dogs)^67^ No association in cats^33^ ↑tBilirubin ↑Mortality (dogs)^4^ No association (dogs, cats)^34^, ^42^, ^66^	Hepatic dysfunction may also affect coagulation
↓Cholesterol	Unknown^68^	↑Mortality, multiorgan dysfunction syndrome, and hospital acquired infection in septic humans^68^ ↑Mortality in dogs with sepsis (all cause)^69^ ↑Mortality in dogs with parvovirus^70^	Functions^68^: Integral part of cell membranes; regulates surface receptors, permeability and fluidity Immunomodulatory properties Binds endotoxin and other toxins preventing toll‐like receptor activation Actions of oxidized derivatives (oxysterols) Vitamin D, hormone and bile acid precursor
↑Creatinine	AKI	AKI at time of presentation associated with high risk of mortality (dogs)^40^	12.3%–40.3% incidence of AKI in SP dogs^40^, ^43^
↓iCa^++^	Relative hypoparathyroidism Vitamin D deficiency or resistance Hypomagnesemia Iatrogenic (NaHCO_3_, citrate anticoagulant or fluid administration)^71^	↑Mortality in dogs with sepsis (all cause)^72^ Common in cats with SP but not associated with mortality^73^	May require supplementation Total calcium should not be substituted for iCa^++^ in dogs or cats^74^, ^75^ Many relevant functions: Vasomotor tone Cardiac contractility Gastrointestinal motility Coagulation cofactor^71^

Abbreviations: AKI, acute kidney injury; SP, septic peritonitis.

SP can lead to wide‐ranging coagulation abnormalities. Disseminated intravascular coagulation (DIC), thrombosis and multiple organ dysfunction syndrome (MODS) are common complications that may occur independently or concurrently.^43^ Preoperative testing identifies patients needing coagulation support, provides prognostic information and informs decisions regarding postoperative thromboprophylaxis.

A 2013 clinical study of SP dogs found complex coagulation derangements.^44^ Antithrombin III (ATIII) was low for all dogs (53% ± 9% survivors, 32% ± 16% non‐survivors; reference range: 65%–145%), and protein C (PC) was low in all non‐survivors (49% ± 26%; reference range: 75%–135%). Similar to human studies, pre‐ and postoperative ATIII and PC levels were associated with survival in this population.^44^, ^76^ Specifically, ATIII activity >41.5% preoperatively and >22.5% postoperatively, and PC activity >60% preoperatively and >49% postoperatively, were each predictive of survival.^44^ In addition to anticoagulant activity, ATIII and PC possess anti‐inflammatory properties.^77^ Higher ATIII and PC in survivors may reflect less severe DIC, however their activity also impacts the underlying inflammatory state driving sepsis and its complications. Activated partial thromboplastin time (aPTT) was prolonged in non‐survivors.^44^ Thromboelastography (TEG) revealed shortened R time in all patients with SP, and otherwise hypercoagulable or largely normal TEG tracings.^44^ Patients with hypercoagulable tracings were more likely to survive. The authors suggest that survivors had a compensated hypercoagulable phenotype, while non‐survivors had a decompensated phenotype characterized by lower ATIII and PC levels and a deficiency of procoagulant factors. This interpretation is supported by a study showing that dogs in DIC with hypercoagulable TEG tracings had improved survival compared to those with hypocoagulable TEGs.^78^

### 2.2 Imaging

Peritoneal effusion hampers radiograph interpretation, but free gas, radiopaque foreign bodies, or uroliths may still be visualized. In one canine study,^79^ 57% of patients with bowel perforation had pneumoperitoneum identified on radiographs. Spontaneous pneumoperitoneum is most commonly attributed to GIT rupture. In dogs, urinary bladder rupture, lung rupture with diaphragmatic hernia,^80^ ruptured splenic abscess,^81^ hepatic abscess,^82^ and idiopathic pneumoperitoneum have been described.^83^ Pneumoperitoneum also occur with penetrating injury, including laparotomy, where postoperative pneumoperitoneum may last 4–5 weeks.^84^, ^85^

Digital radiography can detect as little as 0.5 mL of air, seen most often on lateral projections,^86^ especially in the left lateral view.^87^ Sensitivity is improved by placing the patient in left lateral recumbency for several minutes prior to a horizontal beam radiograph.^88^ Air accumulates under the ribs at the high point of the abdomen. Standing lateral views may also show free gas in the dorsocaudal abdomen.^88^

Point of care ultrasound (POCUS) is commonly used to identify free abdominal fluid and often incorporated into initial patient assessment.^89^ Small amounts of peritoneal fluid are more reliably detected with ultrasound (≥6.6 mL/kg) than radiographs (≥8.8 mL/kg).^90^ Similar to humans, POCUS reliably detected free abdominal fluid in dogs and cats with recent trauma compared to the gold standard of computed tomography (CT).^91^ Ultrasound may be even more sensitive at detecting small volumes of free air, reliably identifying 0.4 mL of air in beagles.^92^ Global Focused Assessment with Sonography in Trauma (GlobalFAST) is recommended when SP is suspected to detect fluid in thoracic and abdominal cavities, assess lung pathology and evaluate intravascular volume.^93^

Complete abdominal ultrasound performed by trained ultrasonographers may yield additional information. Studies evaluating preoperative ultrasound report variable accuracy, likely due to variations in ultrasonographer experience, operator and patient factors and available technology.^94^ In a recent study comparing ultrasonographic to surgical or necropsy findings in 94 dogs and cats with SP, SP was identified by ultrasound in only 56.3%, although the underlying causative lesion was identified in 79.3%.^95^ Ultrasound was better at identifying lesions in the GIT (68% accuracy) than hepatobiliary (27%) or other abdominal organs (31%).^95^ Notably, cytology was the most common means of diagnosis of SP in this population.^95^ Earlier studies report 64%–86.9% agreement between preoperative ultrasound and surgical findings, with gastrointestinal perforation missed in at least one patient in each study.^94^, ^96^, ^97^ The likelihood of detecting clinically relevant ultrasound findings should be weighed against the risk of delays to surgical source control.

Ultrasound may have a role in identifying intestinal surgical site dehiscence (ISSD). Residual peritoneal air does not significantly impair visualization,^67^ and enterotomy and enterectomy sites were identified postoperatively in 96%–100% of dogs and cats.^48^ Ultrasonographic findings are described for dogs after uncomplicated intestinal surgery^98^, ^99^ and with ISSD.^79^ In dogs with confirmed ISSD after handsewn enterectomy, experienced ultrasonographers identified reverberation artifact crossing the intestinal wall in the 11 reported dehiscence sites.^30^ Radiologists or radiology residents identified intestinal wall discontinuity in seven dogs in a study of 114 dogs and cats who underwent intestinal surgery.^48^ Leakage at the surgical site was confirmed intraoperatively in six dogs, while one dog did not undergo surgery and remained in good condition.^48^ Wall thickening, ileus and corrugation were common in all patients, and the absence of local effusion and steatitis was associated with uncomplicated recovery.^48^

Computed tomography is commonly used to investigate acute abdominal pain in humans, and reliably identifies a wide range of intraabdominal pathology.^100^ Rapid image acquisition allows awake or minimally sedated CT in fragile patients.^101^, ^102^ Comparison of radiography, ultrasonography, contrast‐enhanced ultrasonography (CEUS), and contrast‐enhanced multidetector CT (CE‐MDCT) in dogs with acute abdominal signs demonstrated that CE‐MDCT correctly identified the need for surgical intervention in the nine patients, while survey radiography and B‐mode ultrasonography detected this in only eight. Hepatic lesion cytology was required to identify the need for surgery in two hepatic abscess patients.^101^ CEUS detected perfusion deficits not apparent on CE‐MDT^101^; however, cost and stability of contrast media limit its widespread use.^103^ CT detects gastrointestinal foreign body obstructions^104^ and wooden migrating foreign bodies,^105^, ^106^ and may be preferred in larger patients.^102^ However, in seven cases of SP secondary to grass awn migration, only one was suspected to have a grass awn on CT.^11^

### 2.3 Analysis of peritoneal effusion

Peritoneal effusion is collected with ultrasound guidance, or using a four‐quadrant technique, ideally post‐imaging. If fluid is inadequate or inaccessible, diagnostic peritoneal lavage (DPL) can be considered, although quantitative tests will be altered on these samples. Abdominocentesis and DPL techniques have been described.^107^ Sensitivity and specificity of various analytes are presented in Table 2.

**TABLE 2:** Fluid analysis for the diagnosis of SP in dogs and cats.

Variable	Species	Result	Sensitivity	Specificity
Effusion WBC	Dog	Neutrophils>19.88 × 10^3^/μL^108^	80.3%	71.8%
	Dog	TNCC >17 130 × 10^3^/μL^108^	81.6%	65.8%
	Dog	TNCC >13 000 × 10^3^/μL^46^	86%	100%
	Cat	TNCC >13 000 × 10^3^/μL^46^	100%	100%
Cytology	Dog	Bacteria^109^	80%	100%
	Cat	Bacteria^109^	50%	100%
	Mammals	Cytologic interpretation^110^	82.8%	100%
	Dog, Cat	Intracellular bacteria^111^	100%	100%
	Dog	Intracellular bacteria^46^	71%	100%
	Cat	Intracellular bacteria^46^	100%	100%
Glucose	Dog	WB‐effusion gluc >37^112^	89.5%	66.7%
	Dog	Effusion glucose <50 mg/dL^46^	57%	100%
	Dog	WB‐effusion gluc >20^46^	100%	100%
	Cat	WB‐effusion gluc >20^46^	86%	100%
POC glucometer	Dog	WB‐effusion gluc >20^113^	41.2%	100%
	Dog	Plasma‐effusion gluc >20^113^	88.2%	80%
	Dog	Plasma‐effusion gluc >38^113^	88.2%	100%
Lactate	Dog	Effusion lactate >4.2 mmol/dL^112^	72.2%	84.2%
	Dog	Effusion lactate >2.5 mmol/dL^111^	100%	91%
	Dog	WB‐effusion lactate <−1.5^111^	88%	91%
	Dog	WB‐effusion lactate <−2.0^111^	63%	100%
	Dog	WB‐effusion lactate <−2.0^46^	100%	100%
	Cat	Effusion lactate >2.5 mmol/L^111^	67%	67%
	Cat	WB‐effusion lactate <−0.5^111^	78%	78%

Abbreviations: gluc, glucose; POC, point‐of‐care; SP, septic peritonitis; TNCC, total nucleated cell count; WB, whole blood; WBC, white blood cell count.

SP results in highly cellular, high protein effusion.^114^ Total nucleated cell count (TNCC) or neutrophil count may be useful in discriminating septic from non‐septic effusions.^46^, ^108^ Sensitivity of cytology to detect SP is 80%–100%, with 100% specificity in dogs^46^, ^109^, ^110^, ^111^ but only 50% in cats.^109^ Cytologic evaluation with May‐Grunwald Giemsa (MGG) was more likely to detect bacteria than Gram stain.^109^ Most culture positive, cytology negative samples grew *Pasteurella* spp. or small or filiform anaerobic Gram‐negative bacteria.^109^ Samples submitted for diagnostic confirmation should include slides prepared at the time of sampling, as storage results in decreased TNCC and individual cell counts, and loss of intracellular bacteria.^115^

In dogs presenting to an emergency service with peritoneal effusion, effusion glucose was significantly lower in septic effusions than non‐septic effusions, and was at least 20 mg/dL lower than blood glucose in septic effusions.^46^ A subsequent study in dogs with systemic inflammatory response syndrome (SIRS) and peritoneal effusion found 89.5% sensitivity and 66.7% specificity with ≥37 mg/dL whole blood–effusion glucose difference (WB‐EGD),^112^ suggesting that while a high WB‐EGD should raise a strong suspicion of SP, lower WB‐EGD should not be used to rule out SP.

Point of care glucometers overestimate effusion glucose due to low cell mass, leading to low sensitivity to detect SP via WB‐EGD.^10^, ^113^ Using plasma‐effusion glucose difference instead of WB‐EGD improves sensitivity and specificity.^113^

Effusion lactate (EL) and whole blood‐effusion lactate difference (WB‐ELD) also show promise in detecting SP. In dogs with experimental bowel strangulation, EL increased linearly over 24 h.^116^ A study evaluating biomarkers in dogs with SIRS and septic versus non‐septic ascites determined EL was the best discriminator of all variables studied, with EL >4.2 mmol/L yielding 72.2% sensitivity and 84.2% specificity for SP.^112^ WB‐ELD >2.0 mmol/dL correctly identified all septic and non‐septic effusions in dogs in one study^46^; however, a larger study demonstrated that while WB‐ELD < −2.0 mmol/dL was 100% specific, sensitivity was only 63%.^111^ WB‐ELD < −1.5 performed with a sensitivity of 88% and a specificity of 91%.^111^

EL and WB‐ELD appear less useful for detecting septic effusions in cats, although data is limited. EL or WB‐ELD were not significantly different between two cats with SP and five cats without.^46^ In a subsequent study including nine cats with SP and nine without, EL >2.5 mmol/L yielded 67% sensitivity and 67% specificity, while WB‐ELD < −0.5 yielded 78% sensitivity and 78% specificity.^111^

Although most studies described above included patients with neoplasia, clinicians should note that non‐septic neoplastic effusions have been reported to have high lactate and low glucose.^117^

Limited reports suggest that effusion blood gas variables do not reliably discriminate septic from non‐septic effusions in dogs. A study comparing four SP dogs to dogs with adenocarcinoma, glomerulonephritis or hepatic cirrhosis found significantly lower pH and pO_2_, and higher pCO_2_ in dogs with bacterial peritonitis.^118^ A larger study found no significant difference in pCO_2_ or pH between septic and non‐septic effusions in dogs, although pH was significantly lower in cats with SP.^46^

Although cost and availability limit widespread use, biomarkers such as C—C motif chemokine ligand‐2 and cell‐free DNA show promise as prognostic indicators and discriminators between sepsis and non‐sepsis in human and canine patients.^112^, ^119^, ^120^, ^121^, ^122^, ^123^ Blood procalcitonin levels and clearance are commonly used in the diagnosis and management of human sepsis, but are less useful in veterinary patients.^112^, ^124^, ^125^ Similarly, although N‐terminal pro‐C‐type natriuretic peptide (NT‐proCNP) identifies septic humans and offers moderate accuracy in discriminating between dogs with non‐septic SIRS and sepsis,^126^ neither serum nor peritoneal effusion NT‐proCNP levels were useful in detecting SP in dogs.^126^, ^127^

## 3 SURGERY

### 3.1 Surgical source control

Delays of source control of even an hour increase complications and mortality in people.^36^, ^128^, ^129^, ^130^ Conversely, several veterinary studies found no association between SP survival and faster time to surgery.^4^, ^5^, ^33^ Another found longer times to surgery for non‐survivors (8.5 h) than survivors (4.8 h), although inconsistent data prevented statistical comparison.^10^ While preoperative stabilization may reduce anesthetic risk, those with severe, diffuse SP may not improve significantly until surgery is performed.^36^ Based on data limitations in veterinary studies, extensive human studies supporting timely surgery, and the often rapid progression of SP to septic shock and MODS, prioritizing rapid medical and surgical treatment of SP in veterinary patients is a logical course.

Goals of surgical source control include repairing perforations, resecting compromised tissues, and evacuating infected fluid.^129^, ^131^, ^132^ Even when imaging identifies a source of infection, complete abdominal explore is indicated to ensure all sources are found. In people highly compromised by SP, damage control surgery focuses on stopping leaks and resecting non‐viable tissue, postponing anastomosis, enterostomy, and/or closure to future surgery(ies).^128^, ^129^ Temporary cutaneous‐enterostomy allows delay of anastomosis with ongoing bowel evacuation,^128^ but use in veterinary medicine is rare, in part due to management issues and increased costs.^133^

### 3.2 Assessing tissue viability

The GIT is the most common source of SP in small animals.^1^, ^3^, ^4^, ^9^, ^12^, ^13^, ^14^, ^15^, ^23^ Pressure on the intestinal wall compresses microvasculature. Mucosa, the most metabolically active layer, is most susceptible to decreased perfusion, experiencing ischemia at intraluminal pressure of 40 mmHg.^134^ Compromised blood flow leads to intramural edema, further increasing local pressure and decreasing microcirculation.^135^ Full thickness ischemia occurs when intraluminal pressures exceed 80 mmHg, as can happen with a foreign body.^135^

Signs of intestinal non‐viability include lack of arterial pulses and peristalsis, thin wall, failure to bleed when incised, and abnormal color,^135^, ^136^ but there are confounding factors. Reversible hypotension or vasospasm can cause poor arterial pulses.^136^ Peristalsis can occur in grossly ischemic bowel.^136^ Dark red, blue, or purple serosa can be due to traumatic bruising or venous congestion, both of which can resolve favorably.^134^, ^135^, ^136^ Conversely, outer intestinal layers may appear normal even with mucosal necrosis.^137^ Because rapid mucosal repair can occur when circulation is restored, mucosal ischemia is not an absolute indicator for resection.^135^, ^136^

Visual and palpatory assessment of intestinal viability have not proven reliable in human or animal studies.^138^ Prediction by human surgeons of whether an anastomosis would later leak had fairly low sensitivity (38%–62%), specificity (46%–52%), and predictive value (64%).^139^ While palpation and visualization were adequate guides to remove all non‐viable bowel (89% accuracy), this resulted in unnecessary resection of viable tissue in 46% of people.^140^

Various modalities aim to more objectively assess intestinal viability by measuring tissue perfusion, oxygenation, intraluminal pressure, temperature, contractility, or bioimpedance.^135^, ^136^, ^138^ Limitations include conflicting study results, lack of standardization, and accessibility. Fluorescent angiography (FA), in which intravenously‐injected dye is visualized in perfused tissues via an appropriate wavelength of light, is relatively simple, safe, and available in veterinary medicine.^138^ Fluorescent angiography was more predictive of intestinal viability than gross assessment in a clinical human study^140^ and experimental animal studies.^141^, ^142^, ^143^, ^144^, ^145^, ^146^, ^147^ Fluorescein may underestimate viability for intestine that suffered venous, but not arterial, obstruction,^143^, ^144^, ^148^ or overestimate viability due to persistence of dye extravasated from damaged vessels.^142^, ^149^ Near‐infrared fluorescence with indocyanine green is growing in use; advantages over fluorescein include visualization of deeper vessels and faster effect.^146^, ^150^ Research is needed to establish reliable metrics for interpretating FA in clinical veterinary patients.

Because most veterinary intestinal surgery involves relatively focal disease, there is often room to err on the side of some unnecessary resection of viable intestine to ensure resection of all non‐viable bowel. Ancillary modalities may be more routinely indicated when, based on gross assessment, resection would involve excessive length or key regions of intestine (discussed below).

### 3.3 Resection and anastomosis

With the GIT being the most common source of SP, resection and anastomosis is the most common procedure performed in SP patients (Table 3). Risks of anastomosis include dehiscence and stricture. Furthermore, proximal duodenal resection may require cholecystoenterostomy, resecting excessive intestine may cause short bowel syndrome,^152^ ileocecal valve removal increases risk of small intestinal bacterial overgrowth,^153^, ^154^ ileal resection can create the need for supplemental vitamin B12, fat‐soluble vitamins, and taurine,^153^, ^154^ and major colonic resection in dogs may result in longstanding diarrhea.^155^

**TABLE 3:** Canine intestinal anastomoses dehiscence rates relative to presence or absence of preoperative SP and surgical technique.

Study^a^ years, type	Survival rate^b^			Dehiscence rate			Dehiscence rate		Days to DH (range)
	Overall	No DH	With DH	Overall	No preop SP	Had preop SP	HSA	FEESA	
2001–2012 R^56^	71.7% (38/53) in first 2 weeks	78.7% (37/47)^j^	16.7% (1/6)^j^	11.3% (6/53)	11.5% (3/26)^i^	11.1% (3/27)^i^	NA	11.3% (6/53)	4 median (2–5)
2003–2013 R^29^ ^,^ ^c^	NR	NR	33.3% (8/24)	12.1% (24/198)	6.6% (8/122)^h^ 4.2% (2/48) FEESA^i^ 8.1% (6/74) HSA^i^	21.1% (16/76)^h^ 9.7% (3/31) FEESA^k^ 28.9% (13/45) HSA^k^	16.0% (19/119)^h^	6.3% (5/79)^h^	4.7 mean (3–11)
2010–2013 P^31^	85.7% (30/35) in first 2 weeks	100% (30/30)	0% (0/5)	14.3% (5/35)	NR	NR	14.3% (5/35)	NA	3 median (1–5)
2006–2014 R^27^ ^,^ ^d^	87.8% (180/205)	98.3% (173/176)^h^	24.1% (7/29)^h^	14.1% (29/205)	10.3% (16/156)^h^	25% (12/48)^h^	15.7% (21/134)^i^	11.3% (8/71)^i^	3.3 mean (2.6–4)
1994–2016 R^39^	83.3% (75/90) by day 7	NR	NR	10.0% (9/90)	5.5% (4/73)^h^	33.3% (5/15)^h^ 50.0% (2/4) with OA^k^ 8.3% (7/84) without OA^k^	NSD for HSA (71), FEESA (5), and colotomy (14) NSD with or without omentalization (*n* = 6) or serosal patch (*n* = 6)		3.4 mean (2–6)
NR (pre‐2018) R^28^ ^,^ ^e^	NR	NR	31.3% (5/16)	8.9% (16/180)	NR	18.8% (3/16) 60.0% (3/5) HSA^h^ 0% (0/11) FEESA^h^	12.9% (12/93)^h^	4.6% (4/87)^h^	NR
2008–2018 R^151^ ^,^ ^c^	NR	NR	85.7% (6/7)	9.0% (7/78)	2.6% (2/52)^j^	6.4% (5/26)^j^	NA	0% (0/30) with OTSL^h^ 14.6% (7/48) without OTSL^h^	3 median (2–9)
2008–2019 R^51^ ^,^ ^e^	NR	NR	84.6% (11/13)	9.0% (13/144)	5.6% (5/90)^i^	14.8% (8/54)^i^ 19.4% (7/36) FEESA^j^ 5.6% (1/18) HSA^j^	6.0% (3/50)^i^	10.6% (10/94)^i^	4 median (2–17)
2000–2020 R^24^ ^,^ ^f^	80.4% (41/51)	84.4% (38/45)	50.0% (3/6)	11.8% (6/51)	Significantly higher in dogs with preop SP (values NR)		11.8% (6/51)	NA	NR
2011–2024 R^26^ ^,^ ^g^	81.8% (42/55)	86.3% (38/44)^h^	36.4% (4/11)^h^	20.0% (11/55)	2.5% (1/40)^h^	66.7% (10/15)^h^	34.6% (9/26)	NA	NR

Abbreviations: DH, dehiscence; FEESA, functional end‐to‐end stapled intestinal anastomosis; HSA, handsewn anastomosis; NA, not applicable; NR, not reported; NSD, no significant difference; OA, open abdomen; OTSL, oversewn transverse staple line; preop, preoperative; P, prospective study; R, retrospective study; SP, septic peritonitis.

^a^ Only studies with patient populations that extended at least into the last 15 years are included.

^b^ Survival time was to discharge unless otherwise noted.

^c^ Only includes dogs that survived > 72 h postoperatively.

^d^ Only includes dogs that survived the immediate postoperative period.

^e^ Excludes dogs dying for reasons other than dehiscence.

^f^ Includes 33 end‐to‐end and 18 side‐to‐side HSA (NSD in mortality or dehiscence rates).

^g^ Includes 29 enterotomies (2 dehisced) and 26 enterostomies (9 dehisced).

^h^ Significant difference between paired values in adjacent columns of that study.

^i^ No significant difference between paired values in that study.

^j^ Statistical comparison not reported.

^k^ Significant difference between paired values in same column of that study.

Overall dehiscence rates were higher in dogs with preoperative SP (6.4%–66.7%) than without (2.5%–11.5%) (Table 3). Furthermore, dogs with preoperative SP of gastrointestinal origin were 4.4 times more likely to develop postoperative SP than if SP originated from other organs.^15^ Factors detrimental to gastrointestinal healing include mechanical forces, poor incisional apposition, hypoperfusion, hypovolemia, impaired collagen synthesis, excessive collagenases and inflammatory cytokines, microbial dysbiosis, and malnutrition, many of which are common in septic patients.^49^, ^156^, ^157^

Most anastomoses in SP dogs dehisced in the expected 3–5 day window corresponding to the end of the inflammatory/debridement phase of healing, when tissue is weakest (Table 3). However, with reported dehiscence times from 1 to 17 days, it is possible that studies focusing on dogs that survived >72 h postoperatively missed earlier cases of dehiscence, and/or those diagnosing dehiscence at <72 h inadvertently included dogs with persistent SP due to unsuccessful source control.

Regardless of preoperative SP, some studies found a significantly lower dehiscence rate for stapled compared to handsewn anastomoses,^28^, ^29^ while others did not (Table 3).^27^, ^51^ With preoperative SP, dehiscence rates were lower for stapled anastomoses.^28^, ^29^

Stapling is faster than suturing,^27^, ^158^ an advantage in an unstable patient. Adequate staple size is needed to accommodate thick or edematous bowel.^158^, ^159^ Oversewing the transverse staple line is advised to decrease leakage risk.^151^

Due to the colon's poor healing properties and severe consequences of fecal contamination, leak testing (via sigmoidoscopic air distension) is standard in human colorectal surgery and has been shown to reduce dehiscence.^51^, ^160^ Conversely, leak testing for human small intestine is not routine, more often being used when there are serious concerns for healing.^161^ In canine cadaveric intestinal anastomoses, air distension^162^ and intersuture probing with a hemostat^163^ proved more sensitive than saline leak testing. However, the potential impact of these techniques on healing (e.g., causing trauma, disrupting fibrin seal) has not been assessed in live animals, and saline testing remains the standard in veterinary medicine.^51^, ^164^, ^165^

In clinical canine small intestinal anastomoses (hand‐sewn and stapled), there was no correlation between postoperative dehiscence and whether leak test was done, or if it was positive or negative.^51^ Despite the lack of evidence for the value of leak testing in veterinary medicine,^51^, ^163^, ^164^, ^165^ it is still recommended to at least detect subpar surgical closure, especially for novice surgeons.^51^, ^164^

### 3.4 Reinforcement techniques

Reinforcement techniques provide physical and healing support to the GIT. Indications include tissue of uncertain viability that is not readily resectable, areas under tension, and local or systemic factors that increase dehiscence risk, as in SP.^161^, ^166^, ^167^

Omentalization, placement or suturing omentum to the site, is fast and easy. The omentum is an extension of the peritoneum.^168^, ^169^, ^170^ Numerous studies demonstrate the omentum's ability to adhere to sites of inflammation and provide drainage through its extensive vascular and lymphatic networks.^168^, ^171^, ^172^ Omental adhesion also allows direct delivery of blood supply, immune cells, and factors that stimulate cell proliferation, angiogenesis, lymphangiogenesis, neurogenesis, hemostasis, stem cell recruitment, granulation, and healing.^168^, ^169^, ^171^, ^172^ Human studies have mixed results, finding omentalization either decreased^173^, ^174^, ^175^, ^176^, ^177^ or had no impact^178^, ^179^ on the rate of intestinal anastomotic leakage. In experimental anastomoses involving devascularized jejunum in dogs and rabbits, omentalization significantly decreased leakage, and continuity was established between omental and intestinal vessels.^180^, ^181^, ^182^ Veterinarians have observed omental adhesions that appear to have prevented generalized peritonitis in patients with perforated bowel.^39^ Stricture is a potential adverse effect and reported in some experimental cases where omentum was wrapped 360° around devascularized intestine,^180^, ^182^ but the authors did not find reports of omentalization‐related stricture in the clinical human or veterinary literature.

In serosal patching, healthy bowel is sutured to unhealthy bowel, providing blood supply, a fibrin seal against leakage, and more mechanical strength than omentum.^166^, ^167^ Serosal patching of anastomoses in canine cadavers significantly increased leak pressure, even with only 60%–70% coverage.^183^ Serosal patching did not impact the incidence of postoperative SP or mortality in a clinical canine study, although there was a trend towards better outcome and the authors still advised serosal patching be considered in SP patients.^166^ Experimentally, large duodenal defects in dogs patched with jejunum healed, with neomucosa formation across the now intraduodenal jejunal serosa within 6 weeks.^184^, ^185^ In a canine cadaver study, jejunal serosal patches sutured to large duodenal defects did not leak at supraphysiologic pressures.^167^

Of the recent intestinal anastomosis studies in dogs (Table 3), three recorded omentalization and/or serosal patching as being done in some cases, but none reported their impact on outcome.^24^, ^51^, ^151^ A retrospective study of canine colonic surgeries found no significant difference in outcome with omentalization or serosal patching, but each procedure was only used in six of 90 dogs.^39^ It is unknown if the general lack of reporting of these techniques reflects them not being a study focus, incomplete medical records, or actual rarity of use. Considering the potential healing benefits of omentum and serosal patching and paucity of adverse effects reported, it seems prudent to deploy them when healing is of concern, and perhaps to use omentalization routinely (avoiding 360 wrapping) due its particular ease of application.

Patching with porcine small intestinal submucosal (SIS), a biocompatible acellular matrix that provides a scaffold and stimulates repair processes, combined with omentalization, has been used to successfully repair experimentally‐made 7 × 3 cm small intestinal defects in dogs.^186^ In combination with omentalization and/or serosal patching, one of the authors has patched intestinal suture lines of concerning viability with SIS with no apparent adverse effects. Collagen‐based biomaterials such as SIS, as well as fibrin sealants, are both associated with a significant decrease in anastomosis leakage in humans.^186^

In the future, anastomotic healing may be aided by promising treatments such as administering growth factors topically or via gene therapy, “supercharging” (improving local blood supply via microvascular anastomosis), and remote ischemic conditioning (brief, cyclic, limb ischemia and reperfusion, as might be induced by a blood pressure cuff, activates signaling pathways that ultimately improve intestinal healing).^187^, ^188^, ^189^, ^190^, ^191^

### 3.5 Lavage

Potential benefits of lavage in SP include removing microorganisms, foreign material, and inflammatory mediators,^131^, ^192^ decreasing adhesion risk,^192^ patient warming, improved visualization, and identifying the source of bleeding via swirl of rising blood.

In people with SP from perforated diverticulitis sealed by omental or other adhesions, lavage alone can be as beneficial as sigmoidectomy.^193^, ^194^ However, overall evidence of lavage benefits is lacking, and its value is debated.^195^, ^196^ Disadvantages of lavage may include spread of microorganisms, spread and upregulation of proinflammatory mediators, removal of immune cells and cytokines needed for peritoneal defense, promotion of adhesions, and longer surgery time.^14^, ^195^, ^197^, ^198^, ^199^, ^200^ Furthermore, 0.9% saline is not fully biocompatible, with higher sodium and chloride concentrations (~10% and ~50% higher, respectively) and a more acidic pH (5–5.5) than plasma and interstitial fluid.^200^, ^201^, ^202^ In vitro and in vivo studies have demonstrated multiple adverse effects of normal saline on mesothelial cells, including detachment, loss of fibrinolytic activity, increased production of oxygen free radicals and proinflammatory cytokines, and altered receptor‐ligand interactions, even with just 30 min of contact.^192^, ^199^, ^200^, ^201^ Because lavage is added periodically during surgery and thorough suction is not typically done until closing, some peritoneum is likely in contact with lavage for much of the procedure. Ongoing studies may confirm the value of more balanced, buffered lavage solutions such as lactated Ringer's or plasmalyte.^192^, ^201^ Adding antimicrobials to lavage is not advised due to toxic effects on epithelial, endothelial, and mesothelial cells. ^170^, ^192^, ^195^, ^201^

In dogs and cats with SP, pre‐ and post‐lavage cultures often differ in number and species of bacteria isolated. Compared to pre‐lavage, there was a significant decrease in bacterial number and lower likelihood of multidrug resistant (MDR) organisms post‐lavage in one study,^14^ while others found no relation between pre‐ and post‐lavage culture results.^13^, ^197^ All found no survival benefit in dogs that received appropriate empirical antibiotics as indicated by pre‐ or post‐lavage cultures.^13^, ^14^, ^197^ The question of whether to culture pre‐ or post‐lavage, and the true value of routine culture and lavage, remain open.

In veterinary medicine, the standard lavage volume for SP is 200–300 mL/kg. Swayne et al.^197^ traced this back to a source^203^ in which no reference was included. While lavage remains a standard of veterinary SP management, it is prudent to recognize the potential limitations of saline lavage and remove as much fluid as possible to avoid long‐term contact with mesothelial cells. Evacuating residual saline may also facilitate more accurate postoperative ultrasonographic monitoring.

### 3.6 Drainage options

Historically, human patients with severe SP routinely received a planned relaparotomy 24–48 h after the first to reassess tissue viability and peritonitis status, regardless of patient condition.^204^, ^205^, ^206^ However, compared to on‐demand relaparotomy (reoperation only if indicated by clinical condition), planned relaparotomy led to more unnecessary surgeries, longer hospitalization, higher costs, and no significant improvement in peritonitis‐related morbidity or mortality.^205^, ^207^, ^208^, ^209^, ^210^, ^211^ Because intraoperative findings are not predictive of which patients will require relaparotomy, vigilant postoperative monitoring of the previously discussed diagnostics is an important part of an on‐demand strategy.^207^ Development of scoring systems to predict the need for relaparotomy has proved difficult due to low specificity.^212^, ^213^, ^214^ When intraabdominal infection and/or tissue viability are of serious concern during the initial SP surgery, options for ongoing abdominal drainage may be considered.

The World Society for Emergency Surgery did not find strong evidence for open abdomen in people, recommending it be considered only when patient instability requires surgery be kept brief, there is need for deferred intestinal anastomosis or second look for intestinal ischemia, source control was not achieved, or extensive visceral edema precludes closure.^129^ Direct peritoneal resuscitation, in which a hypertonic, glucose‐based peritoneal dialysis solution is placed in the abdominal cavity, has been beneficial in treating ischemic reperfusion injury in people suffering severe trauma, and shows promise for severe septic patients treated with open abdomen.^215^, ^216^, ^217^ The fear a patient might develop intra‐abdominal hypertension (IAH) is not considered sufficient justification for leaving the abdomen open, because IAH is infrequent with SP and can usually be medically managed.^218^ Disadvantages of open abdomen include fluid, electrolyte, and protein imbalances, perpetuation of inflammation, nosocomial infection, need for multiple surgeries, lower fascial closure success, intra‐abdominal abscessation, entero‐atmospheric fistulation, and adhesion formation.^215^, ^216^, ^217^, ^219^, ^220^

In veterinary medicine, open abdomen is most often established by loosely suturing the linea and applying sterile absorptive bandages, requiring intensive management. Historically, survival rates in dogs and cats with SP managed via open abdomen ranged from 42.9% to 100%, generally comparable to contemporaries that had abdominal closure at the initial surgery.^12^, ^15^, ^66^, ^221^, ^222^, ^223^, ^224^ Because open abdomen is typically used for the most severe patients, comparable mortality with closed abdomen may be evidence of open abdomen's benefits, but prospective, controlled studies are needed to determine its true value.

In people, most open abdomens are managed with vacuum assisted closure (VAC).^215^, ^217^, ^225^ Apparent benefits of adding VAC include its pro‐healing effects, easier management, better drainage, decreased risk of ascending infection or evisceration, and decreased fascial retraction.^16^, ^215^, ^220^, ^226^, ^227^, ^228^ Meta‐analysis of studies assessing on‐demand relaparotomy or open abdomen with VAC do not show clear evidence for the value of one over the other.^217^, ^229^ In people treated with open abdomen, combining VAC and continuous traction (via biologic or synthetic mesh sutured to the fascial incision) appears to have the best success in achieving closure, preventing entero‐atmospheric fistula, and overall survival.^217^, ^219^, ^225^, ^230^

In dogs and cats, open abdomen management with VAC was well‐tolerated, with survival rates of 50%–75% after 2–4 days of continuous VAC at −75 to −125 mmHg.^16^, ^227^, ^231^ When SP dogs were randomly assigned to open abdomen or open abdomen plus VAC, the latter was found to be an effective alternative based on comparable survival rates and cost, less leakage, and better peritoneal healing.^16^ VAC times were comparable to or shorter than those for open abdomen (mean: 2–4.4 days, range: 1–9)^16^, ^221^, ^222^ or closure with closed suction drain (mean or median duration 3.6–6 days, range: 2–11).^17^, ^18^, ^232^

Closed‐suction drains provide another option. Compared to open abdomen, benefits of closed‐suction drains include ease of management, ready assessment of fluid quantity and quality, lower risk of ascending infection or evisceration, and not needing a second surgery, while risks include drain obstruction, erosion of bowel or vessels in contact with the drain, and inadequate drainage of peritoneal space.^8^, ^16^, ^17^, ^232^, ^233^, ^234^ In people, closed‐suction drains are controversial and generally reserved for specific conditions (e.g., pancreaticoduodenectomy, colorectal surgery); they are not recommended prophylactically.^157^, ^234^, ^235^, ^236^

Survival rates in dogs and cats with SP managed postoperatively with closed‐suction drain(s) ranged from 52% to 100%.^6^, ^8^, ^17^, ^18^, ^127^, ^232^ Drain obstruction was not reported.^17^, ^18^, ^127^, ^232^ Intracellular bacteria on drain fluid cytology did not correlate with the patient's clinical condition and/or culture results.^18^, ^237^ While cultures taken at drain removal in dogs and cats were often positive, sometimes with different bacteria (often nosocomial) than isolated at surgery, these findings did not appear to impact survival.^18^, ^232^ Even in 10 healthy experimental dogs with closed suction drains after negative abdominal explore, 40% of cultures were positive at drain removal.^237^ In a clinical study, the survival rate of SP dogs with closed suction drains (53%) was significantly lower than without drains (77%); however, dogs with drains had higher illness severity scores, making a direct comparison difficult.^8^

## 4 SUMMARY

Septic peritonitis remains a complex disease, requiring a multifaceted approach to diagnosis and management. Analysis of peritoneal effusion remains the most rapid and reliable diagnostic test to identify SP and underlines the importance of point of care ultrasound in identifying the presence of free abdominal fluid. Advances in diagnostic imaging such as sedated CT scans, CEUS and systematic postoperative abdominal ultrasound can improve the timely detection of SP and identify comorbidities; however, ultimately comprehensive diagnosis and treatment of SP in veterinary medicine require exploratory laparotomy. Advances in surgical techniques such as stapled intestinal anastomoses improve outcomes; however, many surgical decision points such as lavage, leak testing, reinforcement techniques and postoperative drainage lack adequate evidence to make strong recommendations. The substantial variations in survival reported for SP are in part due to the retrospective nature of most veterinary studies and limitations such as incomplete data, small sample size, lack of common protocols, inconsistent timing of diagnostics and treatment relative to disease onset, and unclear impact of euthanasia. Large, prospective, randomized studies are needed to further clarify best practices for SP patients.

## AUTHOR CONTRIBUTIONS

Campbell BG, DVM, PhD, DACVS and O'Marra SK, DVM, DACVECC: Collaborated on the design of the manuscript. Each author wrote substantial portions of the document, critically reviewed the manuscript, and endorsed the final version. The authors are aware of their respective contributions and have confidence in the integrity of all contributions.

## FUNDING INFORMATION

No funding was received for this manuscript.

## CONFLICT OF INTEREST STATEMENT

The authors declare no conflicts of interest related to this report.
